# Epidemiological Pattern and Precipitating Factors of Diabetic Ketoacidosis: A Retrospective Study at King Fahad Specialist Hospital, Buraidah, Saudi Arabia

**DOI:** 10.7759/cureus.111309

**Published:** 2026-06-22

**Authors:** Abdulrahman M Alharbi, Omar K Alsaawi, Naif Y Aljumaah, Abdulrahman A Alkhulayfi, Ibrahim K Alharbi, Essa A Alharbi

**Affiliations:** 1 Department of Endocrinology, King Fahad Specialist Hospital, Buraidah, SAU; 2 Department of Internal Medicine, King Fahad Specialist Hospital, Buraidah, SAU

**Keywords:** diabetic ketoacidosis, epidemiology, saudi arabia, severity predictors, type 1 diabetes mellitus

## Abstract

Background: Diabetic ketoacidosis (DKA) is an acute and severe complication of diabetes that results in increased morbidity and utilization of healthcare resources. Understanding the local patterns and triggers of DKA is important for improving prevention and patient care. This study aimed to assess the epidemiological features and precipitating factors of DKA and evaluate their association with disease severity and time to DKA resolution.

Methods: A retrospective review of 193 patients with DKA admitted to King Fahad Specialist Hospital, Buraydah, Saudi Arabia, from 2023 to 2025 was conducted. Demographic, clinical, biochemical, and etiological variables were analyzed. Associations between DKA severity and resolution time were assessed using the chi-square test, t-test, ANOVA, and multivariate logistic regression.

Results: Most patients were young, with 90 patients (46.6%) aged <18 years. Moderate DKA was the most frequent presentation, affecting 114 patients (59.1%), followed by mild DKA in 51 patients (26.4%) and severe DKA in 28 patients (14.5%). The mean HbA1c level was 11.9%. Missing insulin doses were the main trigger for DKA in 122 patients (63.2%), followed by respiratory tract infections in 21 patients (10.9%) and newly diagnosed diabetes and not on any medications in 16 patients (8.3%). Severe DKA was associated with lower pH, lower bicarbonate, higher anion gap, higher glucose, more pronounced electrolyte abnormalities, and longer resolution times. The etiology of DKA was not significantly associated with severity (p = 0.644) or duration of resolution (p = 0.052); however, infection-related etiologies showed a trend toward longer recovery times.

Conclusion: Although missing insulin doses were the leading trigger for DKA, precipitating etiology did not significantly influence DKA severity or recovery duration. There was no statistically significant relationship between age, chronic diseases, and DKA severity based on the adjusted regression model. DKA severity was the primary determinant of prolonged resolution time. Strengthening adherence, early diagnosis, and risk‑stratified management is essential to reduce the burden of DKA.

## Introduction

Diabetic ketoacidosis (DKA) is a life‑threatening acute metabolic complication of diabetes mellitus, characterized by hyperglycemia, ketonemia, and high anion gap metabolic acidosis resulting from absolute or relative insulin deficiency [1]. Despite advances in diabetes management, DKA remains a major cause of emergency admissions, intensive care utilization, and preventable mortality among individuals with type 1 diabetes mellitus (T1DM) and is increasingly recognized among those with type 2 diabetes mellitus [2,3].

In Saudi Arabia and the wider Middle East, DKA represents a significant clinical and public health concern due to the high prevalence of diabetes and the rising incidence of T1DM among children and adolescents. Studies from Riyadh and other regions have shown that DKA frequently results from preventable causes, particularly missed insulin doses and infections, which contribute to increased morbidity and prolonged hospitalization [4,5]. Research in Saudi children further indicates that severe DKA remains common, often due to delayed recognition of symptoms and limited caregiver awareness of early warning signs [6]. International evidence similarly identifies insulin omission, infection, and poor glycemic control as the predominant triggers of DKA across diverse healthcare systems [1-3].

Global data from Brazil and other regions reinforce that insulin omission, often linked to socioeconomic barriers and limited access to diabetes supplies, is a leading precipitating factor for DKA [7]. These findings underscore that, despite differences in healthcare infrastructure, the underlying triggers of DKA are remarkably consistent worldwide.

DKA poses an even greater challenge in low‑ and middle‑income countries. Studies from Ethiopia and other African settings have shown that infections, delayed hospital presentation, and limited emergency care capacity are major contributors to severe DKA and its complications [8,9]. In South Asian countries such as India, Bangladesh, and Pakistan, DKA continues to cause substantial morbidity and mortality, particularly in rural areas where access to healthcare and diabetes education is limited [10-12]. These regional variations highlight the importance of generating local epidemiological data to guide targeted prevention and management strategies.

Globally, the most common precipitating factors of DKA include insulin omission, infection, and new‑onset diabetes [1-3]. Western and European studies consistently report insulin omission as the leading cause, with infections accounting for approximately one‑third of cases [1-3]. Studies from Saudi Arabia have reported higher rates of insulin nonadherence compared with Western populations, likely reflecting differences in health literacy, socioeconomic status, and continuity of care [4-6]. New‑onset diabetes remains a major contributor to DKA among children and adolescents in the Kingdom, reflecting the increasing burden of T1DM [6].

The severity of DKA varies widely; however, the most severe cases often involve electrolyte disturbances, cerebral edema, and prolonged hospitalization [2]. Evidence from Saudi Arabia, India, and Bangladesh indicates that infection‑related DKA tends to present with more severe metabolic derangements and higher complication rates [4,10,12]. In African and South Asian settings, poor glycemic control, recurrent DKA, and delayed hospital arrival are consistently associated with worse outcomes [8-12]. Recurrence is a key indicator of disease severity, with studies from Ethiopia, India, and Bangladesh showing that repeated DKA episodes are frequently linked to missed insulin doses, financial constraints, and inadequate diabetes education [8-12].

Prognosis varies substantially across regions. High‑income countries report lower mortality rates due to early detection and advanced critical care services [1], whereas low‑ and middle‑income countries experience higher mortality, largely driven by infections, delayed presentation, and limited access to intensive care. Studies from Ethiopia have reported mortality rates ranging from 4% to 16% [8,9], while research from India and Bangladesh shows that severe DKA is associated with significantly higher mortality [10-12].

Numerous studies conducted in Saudi Arabia have investigated DKA. However, most have focused on specific age groups or etiological factors. Investigating this gap is essential to developing preventive strategies and improving clinical outcomes. Hence, this study aimed to evaluate the epidemiological patterns and precipitating factors of DKA and to assess their association with DKA severity and time to resolution among patients admitted to King Fahad Specialist Hospital, Buraidah, Saudi Arabia.

## Materials and methods

Study design and setting

This study was a retrospective single-center analysis conducted at King Fahad Specialist Hospital in Buraidah, Saudi Arabia. The study included patients with T1DM who were admitted with DKA between January 2023 and December 2025. A convenience sampling technique was used, including all available and complete medical records that met the inclusion criteria during the study period.

Inclusion criteria

Eligibility for inclusion was limited to patients who met the American Diabetes Association (ADA) criteria for DKA diagnosis. The diagnosis of DKA was based on the presence of hyperglycemia (blood glucose ≥ 200 mg/dL), metabolic acidosis (arterial pH < 7.3 and bicarbonate < 18 mmol/L), and evidence of ketosis, defined as either ketonemia (≥ 3.0 mmol/L) or ketonuria (urine ketones ≥ 2+ on dipstick) [1].

Exclusion criteria

Patients were excluded if they presented with concurrent hyperglycemic emergencies other than DKA, including hyperosmolar hyperglycemic state; ketosis due to alternative etiologies such as alcoholic or starvation ketoacidosis; or metabolic acidosis from non-DKA causes, including lactic acidosis or uremia. Patients with incomplete hospital records were also excluded, including those lacking essential diagnostic data such as venous blood gas (VBG) results or documentation of the precipitating factor of DKA, as well as patients who were transferred to other hospitals after initial presentation or discharged against medical advice before completion of treatment due to incomplete documentation of in-hospital outcomes.

In total, six patients were excluded from the study: three due to missing VBG results, one due to concurrent metabolic acidosis from uremia, and two who were transferred to other hospitals immediately after initial presentation due to bed unavailability. After exclusions, a total of 193 patients were included in the final analysis.

Data collection

Following approval from the hospital administration and ethics committee, data were retrieved from patient records and entered into Microsoft Excel for analysis. The collected variables included demographic information such as age and sex, comorbidities including hypertension, ischemic heart disease, heart failure, chronic respiratory disease, chronic kidney disease (CKD), liver disease, previous stroke, and smoking status. Clinical presentation data included symptoms such as nausea, vomiting, abdominal pain, blurred vision, drowsiness, and altered mental status, in addition to vital signs, oxygen saturation, and Glasgow Coma Scale score at the time of admission. Laboratory parameters included complete blood count, serum electrolytes, arterial blood gases, and HbA1c levels. Information related to the hospital course, including DKA severity and time to resolution, was also documented.

DKA severity classification and resolution criteria

The severity of DKA was determined according to the ADA criteria based on venous pH and serum bicarbonate levels. DKA was categorized as mild (pH 7.25-7.30 and bicarbonate 15-18 mEq/L), moderate (pH 7.00-7.24 and bicarbonate 10-<15 mEq/L), and severe (pH <7.00 and bicarbonate <10 mEq/L). Resolution of DKA was defined according to ADA criteria as blood glucose <200 mg/dL, serum bicarbonate ≥15 mEq/L, venous pH >7.30, and anion gap ≤12 mEq/L [1].

Statistical analysis

Statistical analysis was performed using IBM SPSS Statistics for Windows, Version 26 (Released 2018; IBM Corp., Armonk, New York, United States). Descriptive statistics are presented as numbers and percentages for categorical variables, whereas means and standard deviations were used to summarize continuous variables. Patients with severe DKA were compared with those with mild or moderate DKA using independent-samples t-tests for metabolic and laboratory parameters. Associations between categorical variables and DKA severity were examined using the chi-square test or Fisher’s exact test when expected cell counts were less than 5. Variables that showed significant associations in bivariate analysis were included in a multivariate logistic regression model to identify independent predictors of severe DKA. The results are presented as adjusted odds ratios (AORs) with 95% confidence intervals (CIs). A normality test was performed using the Shapiro-Wilk test. Furthermore, comparisons between metabolic and laboratory parameters and DKA severity were performed using independent-samples t-tests, whereas comparisons between DKA resolution duration and DKA etiology were conducted using independent-samples t-tests. A one-way ANOVA with variance was used to assess the association between DKA etiology and the duration of DKA resolution. Statistical significance was set at p < 0.05.

## Results

The study reviewed 193 patients with DKA. Table 1 shows that the study population was relatively young, with 90 patients (46.6%) aged < 18 years, while 27 patients (14%) were aged > 30 years. Male and female patients were almost equally represented, 96 (49.7%) and 97 (50.3%), respectively.

**Table 1:** Demographic and clinical characteristics in relation to DKA severity Results are presented as numbers and percentages, N (%). DKA, diabetic ketoacidosis. § p-values calculated using Fisher's exact test. ‡ p-values calculated using the chi-square test. ** Significant at p < 0.05 level.

Study variables	Overall N (%) (n=193)	DKA severity		Statistical test	p-value^§^
		Severe N (%) (n=28)	Mild and moderate N (%) (n=165)		
Age group					
<18 years	90 (46.6%)	20 (71.4%)	70 (42.4%)	Fisher's exact	0.006 **
18 – 30 years	76 (39.4%)	4 (14.3%)	72 (43.6%)	--	--
>30 years	27 (14.0%)	4 (14.3%)	23 (13.9%)	--	--
Sex ^‡^					
Male	96 (49.7%)	14 (50.0%)	82 (49.7%)	χ2=0.001	1.000
Female	97 (50.3%)	14 (50.0%)	83 (50.3%)	--	--
Hypertension					
No	188 (97.4%)	27 (96.4%)	161 (97.6%)	Fisher's exact	0.547
Yes	5 (2.6%)	1 (3.6%)	4 (2.4%)	--	--
Ischemic heart disease					
No	192 (99.5%)	28 (100%)	164 (99.4%)	Fisher's exact	1.000
Yes	1 (0.5%)	0	1 (0.6%)	--	--
Heart failure					
No	192 (99.5%)	27 (96.4%)	165 (100%)	Fisher's exact	0.145
Yes	1 (0.5%)	1 (3.6%)	0	--	--
Chronic kidney disease					
No	189 (97.9%)	28 (100%)	161 (97.6%)	Fisher's exact	1.000
Yes	4 (2.1%)	0	4 (2.4%)	--	--
Asthma					
No	190 (98.4%)	27 (96.4%)	163 (98.8%)	Fisher's exact	0.377
Yes	3 (1.6%)	1 (3.6%)	2 (1.2%)	--	--
Chronic liver disease					
No	191 (99.0%)	28 (100%)	163 (98.8%)	Fisher's exact	1.000
Yes	2 (1.0%)	0	2 (1.2%)	--	--
Smoking					
No	187 (96.9%)	28 (100%)	159 (96.4%)	Fisher's exact	0.596
Yes	6 (3.1%)	0	6 (3.6%)	--	--

The most commonly diagnosed comorbidity was hypertension, seen in five patients (2.6%), followed by CKD in four patients (2.1%) and asthma in three patients (1.6%). When comparing severe DKA with mild or moderate DKA, age group was the only demographic factor significantly associated with severity (p = 0.006). Patients aged <18 years had a significantly higher proportion of severe DKA, with 20 (71.4%) compared with other age groups, 4 (14.3%). There were no significant differences in sex (p = 1.000), hypertension (p = 0.547), CKD (p = 1.000), or other comorbidities (p > 0.05).

**Figure 1:**
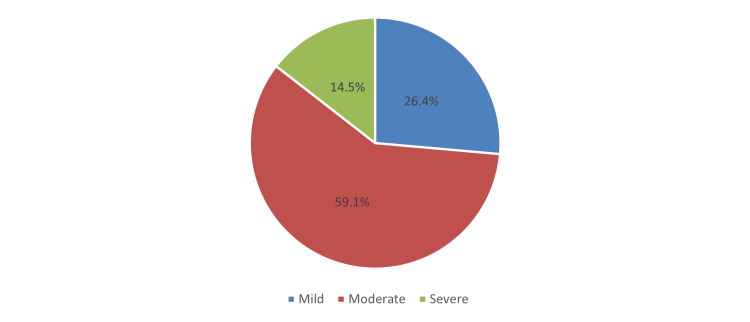
Severity of diabetic ketoacidosis

As shown in Figure 1, moderate DKA was the most common, accounting for 114 (59.1%) of cases, followed by mild DKA, 51 (26.4%), and severe DKA, 28 (14.5%). This indicates that although most patients had significant metabolic issues, only a small proportion developed severe DKA.

Table 2 shows the clinical manifestations in the patients. The most common gastrointestinal symptom was nausea, observed in 154 (79.8%) patients, followed by vomiting in 140 patients (72.5%) and abdominal pain in 138 patients (71.5%). Most patients, 190 (98.4%), had no impairment on the Glasgow Coma Scale. The mean systolic blood pressure, diastolic blood pressure, heart rate, respiratory rate, temperature, and oxygen saturation values were 118.8, 73.8, 100.5, 19.9, 36.9, and 98.3, respectively. No significant relationship was found between nausea, vomiting, abdominal pain, drowsiness, and DKA severity (p > 0.05). In contrast, patients with severe DKA had higher systolic blood pressure (125.8 vs. 117.8 mmHg, p = 0.008), higher heart rate (111.9 vs. 98.5 bpm, p < 0.001), and higher respiratory rate (21.0 vs. 19.7 breaths/min, p = 0.008). The results showed that severe metabolic acidosis triggers a physiological stress response. Temperature, oxygen saturation, and Glasgow Coma Scale scores were similar between the groups (p > 0.05). Thus, vital sign abnormalities, not symptoms, were the key clinical indicators distinguishing severe from mild or moderate DKA.

**Table 2:** Clinical manifestations in relation to DKA severity Results are presented as numbers and percentages, N (%) for categorical variables, or mean ± SD for continuous variables. DKA, diabetic ketoacidosis; AMS, altered mental status; GCS, Glasgow Coma Scale; SBP, systolic blood pressure; DBP, diastolic blood pressure; HR, heart rate; RR, respiratory rate; SPO2, oxygen saturation. § p-values calculated using Fisher's exact test. ‡ p-values calculated using the chi-square test. † p-values calculated using the independent sample t-test. ** Significant at p < 0.05 level.

Study variables	Overall N (%) (n=193)	DKA severity		Statistical test	p-value^§^
		Severe N (%) (n=28)	Mild and moderate N (%) (n=165)		
Nausea ^‡^					
No	39 (20.2%)	5 (17.9%)	34 (20.6%)	χ2=0.112	1.000
Yes	154 (79.8%)	23 (82.1%)	131 (79.4%)	--	--
Vomiting ^‡^					
No	53 (27.5%)	7 (25.0%)	46 (27.9%)	χ2=0.100	0.823
Yes	140 (72.5%)	21 (75.0%)	119 (72.1%)	--	--
Abdominal pain ^‡^					
No	55 (28.5%)	7 (25.0%)	48 (29.1%)	χ2=0.197	0.822
Yes	138 (71.5%)	21 (75.0%)	117 (70.9%)	--	--
Blurring vision					
No	186 (96.4%)	27 (96.4%)	159 (96.4%)	Fisher's exact	1.000
Yes	7 (3.6%)	1 (3.6%)	6 (3.6%)	--	--
Drowsiness					
No	174 (90.2%)	25 (89.3%)	149 (90.3%)	Fisher's exact	0.743
Yes	19 (9.8%)	3 (10.7%)	16 (9.7%)	--	--
AMS					
No	191 (99.0%)	28 (100%)	163 (98.8%)	Fisher's exact	1.000
Yes	2 (1.0%)	0	2 (1.2%)	--	--
GCS					
No impairment	190 (98.4%)	27 (96.4%)	163 (98.8%)	Fisher's exact	0.377
Mild impairment	3 (1.6%)	1 (3.6%)	2 (1.2%)	--	--
	Mean ± SD	Mean ± SD	Mean ± SD		p-value ^†^
SBP	118.8 ± 13.5	125.0 ± 16.3	117.8 ± 12.7	t=2.684	0.008 **
DBP	73.8 ± 9.89	76.4 ± 9.49	73.4 ± 9.92	t=1.506	0.134
HR	100.5 ± 18.0	111.9 ± 20.5	98.5 ± 16.9	t=3.738	< 0.001 **
RR	19.9 ± 2.25	21.0 ± 2.35	19.7 ± 2.18	t=2.708	0.008 **
Temperature	36.9 ± 0.34	36.9 ± 0.36	36.9 ± 0.33	t=-0.931	0.353
SPO2	98.3 ± 1.53	98.4 ± 1.29	98.2 ± 1.57	t=0.558	0.578

**Figure 2:**
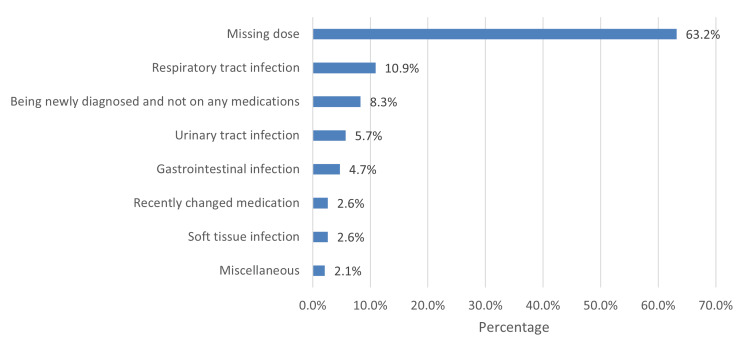
Etiology of diabetic ketoacidosis

As seen in Table 3, missing insulin doses were the leading cause of DKA seen in 122 patients (63.2%), followed by respiratory tract infection in 21 patients (10.9%) and being newly diagnosed and not on any medications in 16 patients (8.3%). Other etiologies were less frequent in patients, including recently changed medication seen in five patients (2.6%) and soft-tissue infections in five patients (2.6%) (Figure 2). The mean duration of DKA resolution was 15.7 ± 11.0 hours. Medication nonadherence and infection patterns were similar across severity groups, and no etiology category was associated with DKA severity (p = 0.644). In contrast, results comparing DKA resolution time showed that patients with severe DKA required substantially longer treatment courses, with a mean resolution time of 26.6 ± 13.2 hours, compared with 13.9 ± 9.49 hours among those with mild and moderate DKA. This difference was statistically significant (p < 0.001), indicating that although the precipitating cause does not predict severity, severe DKA is consistently associated with a prolonged metabolic recovery period. Overall, these findings suggest that etiology alone does not predict severity; however, once severe DKA occurs, it is associated with a significantly extended treatment course.

**Table 3:** Relationship between etiology, duration, and DKA severity Results are presented as numbers and percentages, N (%). DKA, diabetic ketoacidosis; MI, myocardial infarction. § p-values calculated using Fisher's exact test. † p-values calculated using an independent sample t-test. ** Significant at p < 0.05 level.

Study variables	Overall N (%) (n=193)	DKA severity		Statistical test	p-value ^§^
		Severe N (%) (n=28)	Mild and moderate N (%) (n=165)		
Etiology of DKA ^‡^					
Missing dose	122 (63.2%)	16 (57.1%)	106 (64.2%)	Fisher's exact	0.644
Being newly diagnosed and not on any medications	16 (8.3%)	2 (7.1%)	14 (8.5%)	--	--
Respiratory tract infection	21 (10.9%)	6 (21.4%)	15 (9.1%)	--	--
Gastrointestinal infection	9 (4.7%)	1 (3.6%)	8 (4.8%)	--	--
Urinary tract infection	11 (5.7%)	1 (3.6%)	10 (6.1%)	--	--
Recently changed medication	5 (2.6%)	1 (3.6%)	4 (2.4%)	--	--
Soft tissue infection	5 (2.6%)	1 (3.6%)	4 (2.4%)	--	--
Miscellaneous	4 (2.1%)	0	4 (2.4%)	--	--
Duration of DKA resolution in hours (mean ± SD) ^†^	15.7 ± 11.0	26.6 ± 13.2	13.9 ± 9.49	t=6.145	< 0.001 **

Table 4 explores whether the underlying etiology of DKA influences the duration of DKA resolution. Patients with infection-related DKA, particularly gastrointestinal and urinary tract infections, had the longest mean resolution times, whereas those newly diagnosed or with miscellaneous causes had the shortest. Intermediate recovery times were observed in cases involving missed doses, recent medication changes, or respiratory or soft-tissue infections. Although these patterns suggest that infectious etiologies may prolong metabolic stabilization, the overall difference across groups did not reach statistical significance (p = 0.052).

**Table 4:** Association between etiology of DKA and duration of DKA resolution Results are presented as mean ± SD. DKA, diabetic ketoacidosis. § p-values calculated using the one-way ANOVA test.

Factor	Duration of DKA resolution in hours Mean ± SD	Statistical test	p-value ^§^
Etiology of DKA ^‡^			
Being newly diagnosed and not on any medications	11.5 ± 4.53	F=2.039	0.052
Gastrointestinal infection	23.4 ± 12.1	--	--
Missing dose	15.1 ± 8.56	--	--
Recently changed medication	14.0 ± 6.16	--	--
Respiratory tract infection	16.3 ± 15.4	--	--
Soft tissue infection	15.2 ± 9.12	--	--
Urinary tract infection	23.8 ± 24.6	--	--
Miscellaneous	12.0 ± 4.08	--	--

Table 5 shows that, after adjusting for sex, nausea, and vomiting, age group did not emerge as a significant factor for DKA severity (p > 0.05).

**Table 5:** Multivariate logistic regression analysis to determine the independent significant risk factors of DKA severity Adjusted for sex, nausea, and vomiting. DKA, diabetic ketoacidosis; AOR, adjusted odds ratio; CI, confidence interval. ** Significant at p < 0.05 level.

Factor	AOR	95% CI	p-value
Age group			
< 18 years	Ref		
18 – 30 years	0.586	0.179 – 1.917	0.377
> 30 years	3.262	0.750 – 14.180	0.115

Table 6 shows that patients with DKA demonstrated marked metabolic derangements, reflected by a low mean pH (7.17 ± 0.11), elevated anion gap (21.2 ± 5.93 mmol/L), and markedly high random blood sugar levels (465.4 ± 151.20 mg/dL). Laboratory parameters, including white blood cell count, platelet count, and electrolytes, also showed considerable variability across the cohort. When comparing severity groups, patients with severe DKA exhibited significantly more profound metabolic abnormalities. Severe cases had markedly lower pH (6.98 vs. 7.20; p < 0.001), lower bicarbonate levels (6.1 ± 2.49 mmol/L vs. 12.7 ± 3.35 mmol/L; p < 0.001), and substantially higher anion gap (26.6 mmol/L vs. 20.3 mmol/L; p < 0.001), confirming greater acidosis and metabolic disturbance. Random blood sugar was also significantly higher in severe DKA (557.0 mg/dL vs. 449.9 mg/dL; p < 0.001). Inflammatory markers differed as well, with severe cases showing higher white blood cell counts (17.9 ×10⁹/L vs. 12.0 ×10⁹/L; p = 0.016). Hemoglobin and albumin levels did not differ significantly between groups (p > 0.05). Platelet counts were higher in severe DKA (421.5 ×10⁹/L vs. 368.8 ×10⁹/L; p = 0.038), whereas electrolyte comparisons showed significantly higher potassium (5.0 mmol/L vs. 4.6 mmol/L; p = 0.018), higher sodium (134.6 mmol/L vs. 132.3 mmol/L; p = 0.012), and higher chloride levels (102.0 mmol/L vs. 99.3 mmol/L; p = 0.035) among severe cases. HbA1c levels were similar across groups (p = 0.893). Overall, these findings indicate that severe DKA is characterized by more severe acidosis, a higher anion gap, greater hyperglycemia, elevated inflammatory markers, and notable electrolyte shifts.

**Table 6:** Metabolic and laboratory characteristics Results are presented as mean ± SD. DKA, diabetic ketoacidosis; pH, potential of hydrogen; AG, anion gap; RBS, random blood sugar; WBC, white blood cell; HB, hemoglobin; PLT, platelet; ALB, albumin; K, serum potassium; NA, sodium; CL, serum chloride; HBA1c, glycated hemoglobin. § p-values calculated using an independent sample t-test. ** Significant at p < 0.05 level.

Variable	Overall mean ± SD	DKA severity		t-test	p-value ^§^
		Severe mean ± SD	Mild and moderate mean ± SD		
pH	7.17 ± 0.11	6.98 ± 0.10	7.20 ± 0.08	-13.315	< 0.001 **
AG	21.2 ± 5.93 mmol/L	26.6 ± 7.10 mmol/L	20.3 ± 5.21 mmol/L	5.563	< 0.001 **
RBS	465.4 ± 151.20 mg/dL	557.0 ± 161.60 mg/dL	449.9 ± 144.20 mg/dL	3.572	< 0.001 **
WBC	12.9 ± 11.90 ×10⁹/L	17.9 ± 6.51 ×10⁹/L	12.0 ± 12.40 ×10⁹/L	2.439	0.016 **
HB	14.4 ± 2.11 g/dL	14.1 ± 3.32 g/dL	14.4 ± 1.85 g/dL	-0.765	0.445
HCO3	11.7 ± 3.99 mmol/L	6.1 ± 2.49 mmol/L	12.7 ± 3.35 mmol/L	-9.965	< 0.001 **
PLT	376.5 ± 124.20×10⁹/L	421.5 ± 118.10×10⁹/L	368.8 ± 123.90 ×10⁹/L	2.091	0.038 **
ALB	44.7 ± 5.55 g/L	45.8 ± 5.80 g/L	44.5 ± 5.51 g/L	1.134	0.258
K	4.7 ± 0.73 mmol/L	5.0 ± 1.07 mmol/L	4.6 ± 0.64 mmol/L	2.390	0.018 **
NA	132.6 ± 4.59 mmol/L	134.6 ± 6.86 mmol/L	132.3 ± 4.01 mmol/L	2.524	0.012 **
CL	99.7 ± 6.21 mmol/L	102.0 ± 9.36 mmol/L	99.3 ± 5.44 mmol/L	2.125	0.035 **
HbA1c	11.9 ± 3.64 %	11.8 ± 2.46 %	11.9 ± 3.81 %	-0.134	0.893

## Discussion

This study examined the epidemiological patterns, precipitating factors, and determinants of disease severity among patients admitted with DKA at King Fahad Specialist Hospital in Buraidah. Approximately 90 (47%) of our cohort were aged <18 years, consistent with global and regional data showing that DKA is more common in younger patients with T1DM [1,2]. This pattern is also observed in Saudi Arabia and other countries where many DKA admissions occur among adolescents and young adults [13,14]. Research from India and Ethiopia also found that younger age groups often present with DKA, generally due to delayed diagnosis or improper treatment [10,15].

The proportion of patients with severe DKA in this study was 14.5% and was lower than the 25-40% reported in several regional and international studies [4,7]. Similar results were found in Croatia, where most patients had moderate DKA; severe cases were less common but linked to serious metabolic problems [16]. The lower number of severe DKA cases in our study may be due to earlier presentation, better triage, or greater community awareness of DKA symptoms.

Gastrointestinal symptoms, particularly nausea, vomiting, and abdominal pain, were the most frequently observed presenting features. These findings are consistent with the classic clinical features of DKA described in international literature [1]. Similar patterns have been reported in India and Croatia, where gastrointestinal complaints constitute the primary symptoms of DKA [10,16]. In this study, abnormal vital signs such as tachycardia and tachypnea were closely associated with severe DKA, reflecting the body's response to metabolic acidosis. Similar findings from Ethiopia and India reported that severe DKA often involves significant changes in breathing and heart rate due to compensatory mechanisms [10,15].

Insulin omission was the primary precipitating factor seen in 122 (63.2%) of patients, followed by infection. This trend is similar to global findings, in which insulin omission was the most common cause of DKA, especially among adolescents and young adults [9,12]. Studies from Saudi Arabia and India have also shown that insulin omission was the main trigger, often related to psychosocial barriers, poor adherence, and limited diabetes education [4,10]. Infections accounted for 23.8% (46) of patients in our study, which is lower than the 30-50% reported in Western and Asian populations [1,3]. However, infection is a major cause in African settings, where delayed care and limited access to medical services lead to more severe cases [15].

In the multivariate model, age group was not a significant independent predictor of severe DKA after adjusting for sex, nausea, and vomiting. Although adults aged >30 years had a higher AOR than those under 18 years, the wide confidence interval and non‑significant p‑value indicated that this trend was not statistically significant. This result is inconsistent with studies focusing on children, which often report higher severity in children and adolescents [6]. However, studies from Ethiopia and Tunisia have shown that adults may have more severe metabolic problems because their symptoms are recognized later, are less typical, or due to other health issues that exacerbate illness severity [15,17]. The negative association between the time required to resolve DKA and its severity in the regression model is an example of reverse causation because more severe DKA cases naturally require longer treatment. Other studies examining DKA outcomes have reported similar modeling challenges [14].

The biochemical markers in our study showed the expected trends. Severe DKA was associated with lower pH and bicarbonate, a higher anion gap, and more pronounced electrolyte imbalances, which aligns with established pathophysiology and international data [1,3]. Our results are similar to those from Croatia, India, and Ethiopia, where severe DKA is also characterized by deeper acidosis and greater electrolyte disturbances [10,15,16].

Potassium abnormalities are strongly associated with severe DKA and longer recovery time. This finding is consistent with studies conducted in Saudi Arabia and Tunisia, which also identified potassium imbalances as significant indicators of metabolic severity and predictors of delayed recovery [17,18].

The findings regarding the duration of DKA resolution were consistent with those reported by AlWahbi et al. [18], who investigated the factors influencing the time to correction of metabolic parameters in patients with DKA in Saudi Arabia. The study showed that the severity of acidosis and electrolyte problems, especially potassium issues, mainly determined how long it took for patients to recover. Patients with severe biochemical problems require longer periods of insulin and fluid therapy to achieve metabolic stability. This is consistent with the current study's finding that severe DKA cases took nearly twice as long to resolve as mild or moderate cases.

The underlying cause of DKA did not significantly affect the condition's severity, and for most causes, it did not affect the time to resolution. These findings are consistent with those from Croatia and Saudi Arabia, where the time required for metabolic recovery depends mainly on the severity of acidosis and electrolyte disturbances rather than on the trigger of the episode [16,18]. Studies from India and among cancer patients have also shown that clinical severity, rather than the cause, is the main factor in treatment duration and in how patients respond [10,19].

Overall, the severity of metabolic disturbances, particularly potassium imbalance and acidosis, had the greatest impact on the clinical outcomes. The discrepancies between these findings and those reported in other studies are likely attributable to differences in population demographics, access to healthcare, and adherence to treatment protocols. These findings highlight the need to implement targeted patient education, enhance support for treatment adherence, and adopt risk-based management strategies to reduce the burden of DKA in this region.

Limitations

This study had several limitations. As this was a retrospective study, data collection was not standardized or specifically designed for research purposes, limiting control over variables and increasing the susceptibility to bias and confounding factors. Additionally, the retrospective design restricts the ability to establish causal relationships. Moreover, we were unable to assess the behavioral or psychosocial reasons for insulin omission. Because the study was conducted at a single center, the results may not apply to other regions with different populations or healthcare systems. We also did not separate new onset from established diabetes cases, which may have affected how DKA presented and its severity. Furthermore, the relatively small number of severe DKA cases may have limited the statistical power of the multivariate regression analysis to detect independent predictors of severity. Variability in inpatient management protocols and clinician-dependent differences in fluid resuscitation and insulin therapy may have influenced the observed duration of DKA resolution. Lastly, exclusion of patients may have introduced bias; however, the number excluded was small, and the impact on the overall findings is likely limited.

## Conclusions

This study provides a comprehensive overview of the main characteristics, causes, and factors affecting the severity of DKA among patients admitted to King Fahad Specialist Hospital in Buraidah, Qassim, Saudi Arabia. The main reason for DKA was missing insulin doses, indicating ongoing challenges with medication adherence. Infections were not as common as in other countries, but still played a significant role. Furthermore, the study found that certain metabolic factors, including electrolyte abnormalities, pH, and anion gap, were strongly associated with DKA severity and time to resolution. It also showed that the cause of DKA had little effect on recovery time, indicating that severity at admission was the main factor of the disease course. Based on the adjusted regression model, there was no statistically significant relationship between age, chronic diseases, and DKA severity. These findings highlight the need for better strategies to improve medication adherence, early detection, and risk-based management to reduce the burden of DKA in this region.
